# Reproductive Cold Stress in Contrasting Sorghum Genotypes: Is Pollen Fertility Really the Crucial Trait?

**DOI:** 10.1002/pld3.70065

**Published:** 2025-05-04

**Authors:** Luisa Neitzert, Natalja Kravcov, Benjamin Wittkop, Rod Snowdon, Steffen Windpassinger

**Affiliations:** ^1^ Department of Plant Breeding, IFZ Research Center for BioSystems, Land Use and Nutrition Justus‐Liebig University Giessen Giessen Germany

**Keywords:** climate adaptation, cold sensitivity, pollen fertility, reproductive cold tolerance, *Sorghum bicolor*, sorghum hybrid breeding, spikelet fertility

## Abstract

The influence of cold stress during the reproductive phase can lead to substantial yield losses in sorghum. In order to extend cultivation into temperate regions, a better understanding of reproductive cold tolerance is essential for breeding progress. To further elucidate the mechanisms responsible for cold tolerance, a cold‐tolerant and a cold‐sensitive parental line, along with their reciprocal F1 hybrids, were subjected to cold stress at various stages of reproductive development, with a focus on pollen fertility and receptivity of female floral organs. For this purpose, pollen measurements were conducted using impedance flow cytometry, and the *panicle harvest index* was determined post‐maturation. While existing literature primarily attributes reduced pollen fertility as the cause of decreased seed set, this study provides evidence that female floral organs might be more affected than previously assumed. We found that the onset of generative tissue formation until BBCH39 (flag leaf visible) is the most cold‐sensitive developmental stage and that there is no predominance of maternal or paternal effects associated with the inheritance of cold tolerance in reciprocal F1 hybrids. These findings offer valuable insights for the development of cold‐tolerant sorghum varieties to enable cultivation in colder regions and enhance yield stability in temperate climates. Further studies should aim at validating and expanding these findings from the limited number of representative genotypes analyzed in the present manuscript to global sorghum diversity.

## 1 Background

The significance of cold tolerance in sorghum for expanding cultivation into temperate regions is attracting growing scientific and agricultural interest. As population density steadily increases, the availability of fertile soil decreases (Bindraban et al. 2012). Sorghum, one of the world's key cereals for food and feed production, is known for its adaptability to various environmental conditions (Doggett 1988; Berenji and Dahlberg 2004; Zheng et al. 2011; FAO 2024). Originating from semiarid regions of Africa, this crop (Rakshit and Wang 2016) demonstrates high drought tolerance, efficient water and nutrient utilization, resistance to *Diabrotica virigifera* (Oyediran et al. 2004), and the ability to thrive on marginal soils, providing a competitive advantage for future climate changes (Patil 2017). However, cold stress, particularly in temperate regions but also in tropical high‐altitude environments, presents a significant challenge (Pinthus and Rosenblum 1961). Sorghum is not only sensitive in the seedling stage but also exhibits increased susceptibility to cold stress during the reproductive phase, resulting in reduced or even no seed set (i.e., complete yield loss), depending on the severity of the stress and susceptibility of the genotype (Maulana and Tesso 2013; Chopra et al. 2017; Emendack et al. 2021).

Existing literature predominantly emphasizes reduced pollen fertility (Caddel and Weibel 1971; Downes and Marshall 1971) as the primary limiting factor for sorghum cultivation in temperate regions (Maulana and Tesso 2013). However, the extent to which the female floral organ, more precisely the pistil receptivity, is affected by cold stress is less well explored but has been a subject of discussion (Osuna‐Ortega et al. 2003). If the stigma is unable to capture or properly transmit pollen under cold stress, fertilization cannot occur, leading to reduced or completely absent seed formation and severe yield losses, even when fertile pollen is present in sufficient quantities. Because the stigma plays a central role in successful fertilization, understanding its response to cold stress is essential for developing strategies to breed sorghum with improved cold tolerance. A detailed investigation of this aspect could help to establish targeted breeding approaches that consider not only pollen fertility but also stigma receptivity, thereby optimizing reproductive performance in colder climates. Considering the complexity of the traits, juvenile and reproductive cold tolerance can be assumed to be both heterotic and quantitative traits (Schaffasz, Windpassinger, Friedt, et al. 2019; Schaffasz, Windpassinger, Snowdon, et al. 2019; Windpassinger et al. 2015) Alleles involved in reproductive cold tolerance seem to be inherited dominantly, and a sustained breeding progress can be expected due to high phenotypic variation and high heritability (Schaffasz, Windpassinger, Snowdon, et al. 2019).

This work aims to gain a broader understanding of reproductive cold tolerance in sorghum, by unraveling several open questions: (i) What role do the developmental stage of sorghum and the duration of cold stress play; (ii) can reduced seed set be explained by reduced pollen fertility solely; (iii) or is there evidence for a more prominent role of female floral organs; (iv) can we observe a predominance of maternal or paternal cold tolerance effects in reciprocal hybrids.

Altogether, this study could contribute to speeding up the breeding progress toward cold tolerant sorghum with enhanced yield stability in temperate areas.

## 2 Materials and Methods

### 2.1 Plant Material

For the experiments, one cold‐tolerant and one cold‐sensitive inbred line and their reciprocal F1 hybrids were studied. The two inbred lines were selected from a diversity set of *Sorghum bicolor* (*n* = 330) consisting of inbred lines of different origin, utilization and subspecies. Traits such as seed yield and *panicle harvest index* (PHI) under cold conditions were used to evaluate reproductive cold tolerance (Chakrabarty et al. 2021).

**FIGURE 1:**
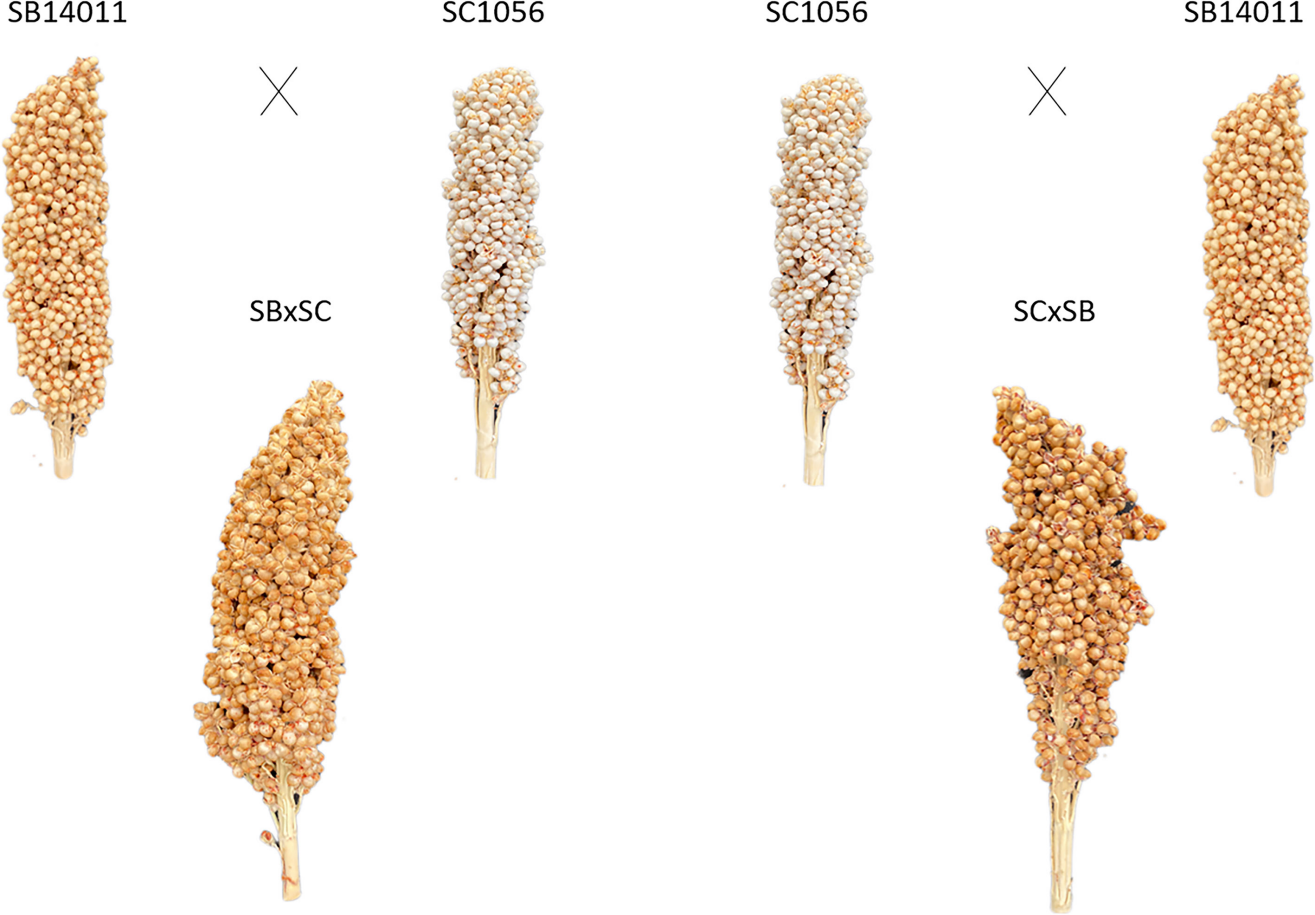
Cold‐tolerant parental line SB14011 and cold‐sensitive parental line SC1056 and their reciprocal F1 hybrids (SB × SC and SC × SB) (grown under optimal conditions [see Table 1]).

Thus, genotype SB14011, a breeding line developed and selected under German conditions, was identified as cold tolerant and SC1056, a conversion line originating from Sudan, as cold sensitive. In addition, reciprocal F1 hybrids, shown in Figure 1, were created by hand emasculation. (Because both inbred lines are restorers in A1 cytoplasm, hand emasculation was the only way to create hybrids among them.)

### 2.2 Defining Growth Stages Based on BBCH Scale

**FIGURE 2:**
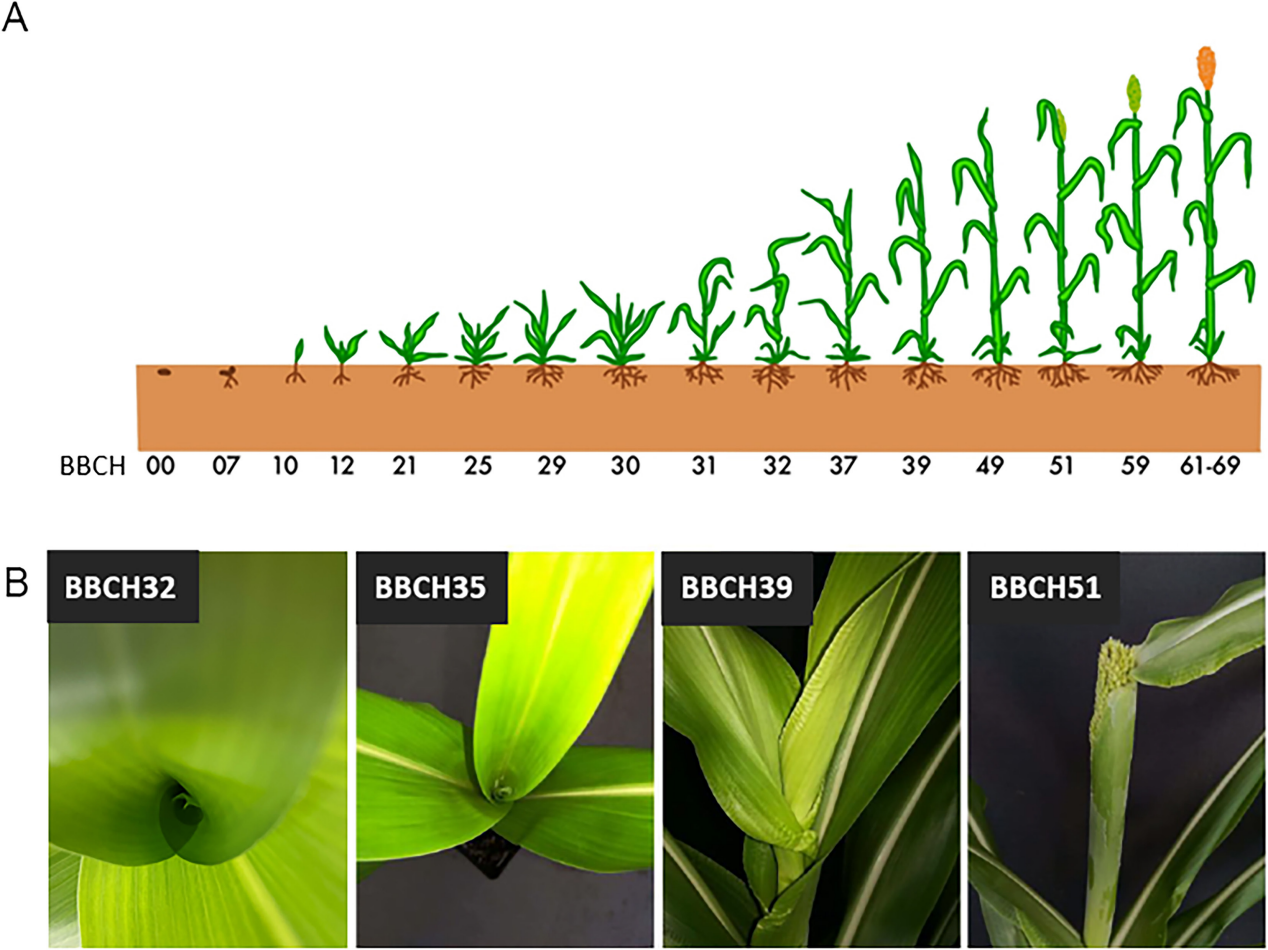
(a) BBCH scale for sorghum in reference to the cereal scale developed by Zadoks et al. (1974). (b) Overview of the developmental stages to be examined—BBCH32: flag leaf not yet visible, seat low down; BBCH35: flag leaf not yet visible, shortly before the appearance of the leaf tip; BBCH39: ligule of the flag leaf visible; BBCH51: emergence of the panicle.

To examine the plants at different developmental stages, the BBCH scale was used, a standardized, detailed coding system for the phenological developmental stages of monocotyledons and dicotyledons. This scale is based on a decimal system, divided into macrostages and microstages. Its structure follows the grain scale developed by Zadoks et al. (1974) in order to avoid major changes to an already well‐established and widely used scale. A graphical overview of the sorghum growth stages is provided in Figure 2a. In the present work, the plants were examined for the influence of cold from BBCH32, BBCH35, BBCH39, and BBCH51 onwards (Figure 2b). At BBCH32, the plant is in the two‐node stage; i.e., the second node is perceptible, at least 2 cm from the first node. At BBCH35, the plant has reached the five‐node stage; i.e., the fifth node is at least 2 cm from the fourth node. The flag leaf is very small at this stage, has its seat relatively far down, and is completely curled up. At stage BBCH39, the flag leaf is now fully developed and has visible ligules. At BBCH51, heading has begun; i.e., the panicle tip emerges from the leaf sheath.

### 2.3 Climate Chamber Experiments

The climate chamber experiment was carried out at the IFZ Research Center for Biosystems at the Justus Liebig University in Giessen, Germany. Both inbred lines and their reciprocal F1 hybrids were tested under controlled cold stress and optimum growth conditions at different stages (BBCH32, BBCH35, BBCH39, and BBCH51) in the reproductive phase. Plants were grown in 17 × 17 × 17 cm pots filled with Fruhstorfer type N soil and were watered and fertilized following good horticultural practice to exclude any other stresses. During emergence and early vegetative growth, all plants were grown under optimum conditions (30°C/13 h during the day and 24°C/11 h at night, with a relative humidity of 60% and metal halide lamps for lighting). For each of the above‐mentioned BBCH stages, randomly selected plants were transferred to a cold stress chamber (25°C/13 h during the day and 7°C/11 h at night, with relative humidity of 60% and LED lighting), where they remained from the respective BBCH stage until the beginning of the grain filling phase (BBCH 71). By this approach, the following two factors are combined: (i) a specific initial phase and (ii) different stress durations.

For each genotype and BBCH stage, three plants were stressed. These individual plants were considered as replicates for statistical evaluation.

#### 2.3.1 Male Versus Female Floral Organ

Transparent Cryovac bags (330 mm × 750 mm, 15 μm) (Sealed Air, Charlotte, NC, USA) were placed over the panicles before flowering to ensure self‐pollination for the considered seed set traits.

**FIGURE 3:**
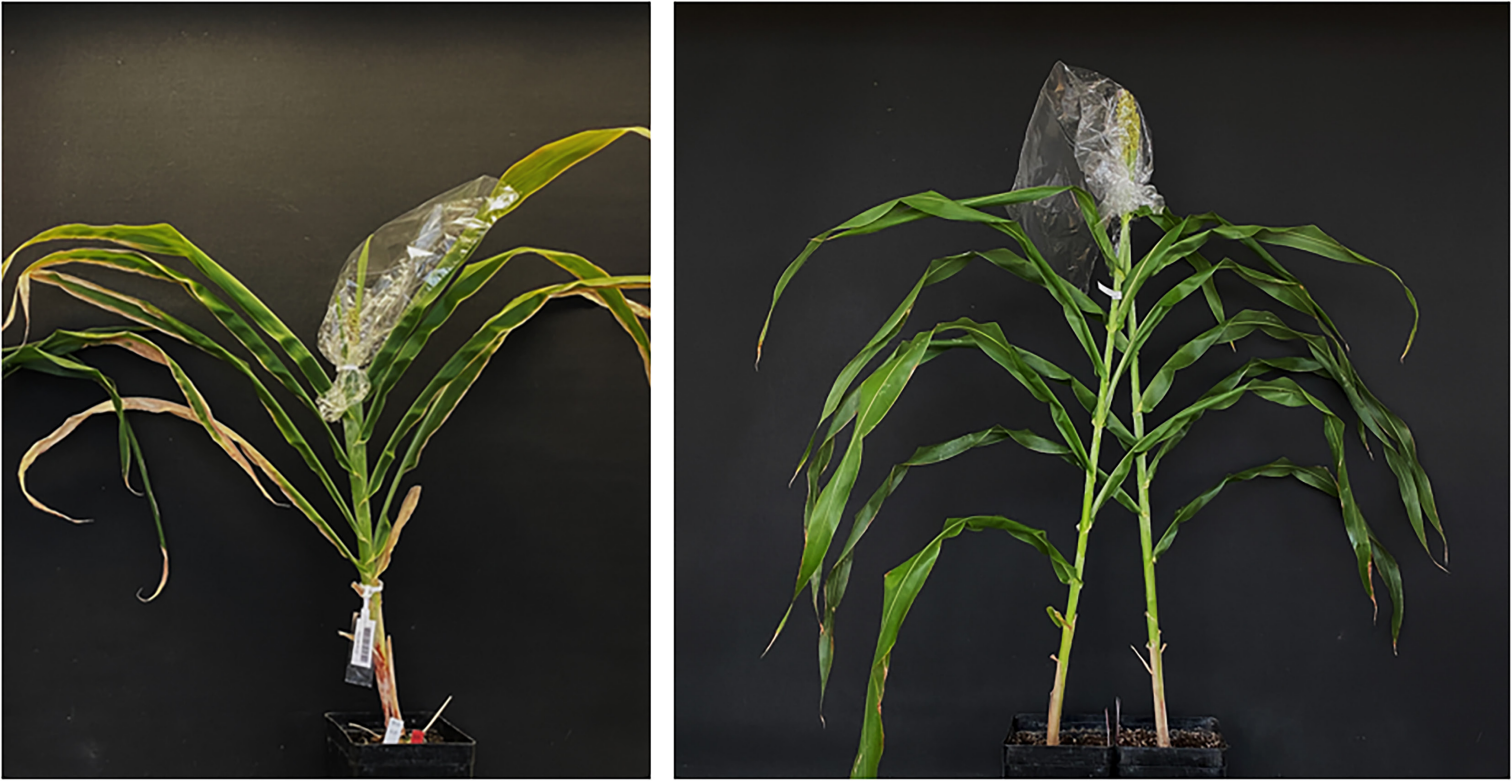
Left: Pollen fertility experiment—individual sorghum plant bagged prior to anthesis to ensure self‐pollination; right: Pollen receptivity experiment—stressed sorghum plant (“female plant”) bagged together with an unstressed pollinator plant (“male plant”) of the same genotype.

To analyze the effect of cross‐pollination, a plant grown under optimal conditions (“male plant”) was packed under the Cryovac bags together with a stressed plant (“female plant”) (25°C/13 h during the day and 7°C/11 h at night, with relative humidity of 60% and LED lighting) of the same genotype shortly before anther emergence throughout the flowering period (Figure 3). Thus, when evaluating the receptivity of the pistil, it can be excluded that sterile pollen led to low PHI. The “female plants” were each stressed at the BBCH stages described above. This experiment was conducted for both inbred lines (susceptible line SC1056 and tolerant line SB14011) and their reciprocal hybrids (SB × SC and SC × SB), in three replicates each.

### 2.4 Pollen Analysis via Amphasys Impedance Flow Cytometry (IFC)

Pollen analyses was conducted using IFC, manufactured by Amphasys AG (Amphasys AG, Root, Switzerland). This technique measures electrical capacity (and hence viability) of a cell, utilizing a small microfluidic chip where the pollen grains flow through and the electric charge of the cells is measured via different radio frequencies (Heidmann et al. 2016). Sample collection and measurements were carried out applying established protocols for sorghum described previously by Schaffasz, Windpassinger, Snowdon, et al. 2019.

### 2.5 PHI

To determine the PHI (Krishnamurthy et al. 2014), at seed maturity the panicles were harvested by cutting them just below the first branches and dried. The total weight of the panicle was then determined before threshing and the pure seed weight after threshing.

Subsequently, the PHI was calculated based on the previously collected data using the following formula (Krishnamurthy et al. 2014):

$$\begin{array}{c} \mathrm{PHI}=\text{grain}\;\mathrm{dry}\;\text{weight}\;\left(i.e.,\text{seed yield}\;\mathrm{per}\;\text{panicle}\right)/\\ \text{panicle}\;\mathrm{dry}\;\text{weight}\;\left(\text{before threshing}\right) \end{array}$$

Consequently, a PHI value of 0 implies absolutely no seed set, while values close to 1 indicate a high seed set. However, even assuming complete spikelet fertility, PHI will be < 1, due to the panicle raw weight. In addition, seed number was measured using seed‐counter Contador (Pfeuffer GmbH, Kitzingen, Germany). All these traits (PHI and seed number) were scored on both stressed and nonstressed plants.

### 2.6 Statistical Analysis

A multifactorial ANOVA was performed for all scored traits, taking into account the independent variables “treatment” and “genotype” as fixed factors to analyze possible interaction effects.

Hence, the ANOVA model applied here followed the general form:

$$\begin{array}{c} Y=\mu +\text{Treatment}+\text{Genotype}\\ \;+\text{Treatment}\times \text{Genotype}+\text{Error} \end{array}$$

where *Y* represents the dependent variable, *μ* is the overall mean, “Treatment” and “Genotype” are fixed main effects, and “Treatment × Genotype” represents the interaction between these factors. The error term accounts for the random variation within the data (residual variance).

Separate one‐way ANOVA analyses were performed for each specific trait to asses possible differences between treatment levels (BBCH32, BBCH35, BBCH39, BBCH51, and control) within a genotype. The independent variable in each analysis was “treatment”, and the dependent variable was the respective trait. These characteristics included the yield parameters PHI and seed yield as well as the pollen parameters percentage of fertile pollen (%), cell concentration (cells/mL), and total number of fertile pollen (fertile pollen/mL) measured with the impedance flow cytometer.

Following each ANOVA, a least significant difference (LSD) post hoc analysis was performed to determine which treatments showed statistically significant differences from each other.

*t*‐tests were performed to analyze significant differences between cross‐pollination and self‐pollination at a specific treatment level. A paired *t*‐test was performed to analyze significant differences between the parental lines SB14011 and SC1056 and their reciprocal F1 hybrids within a given treatment level.

Pearson's correlation coefficients were used to assess the relationship between the pollen traits and the yield parameters.

The statistical analysis and the presentation of the results were carried out in the R version 4.0.5.

## 3 Results

### 3.1 Sensitivity to Cold During the Reproductive Stage

#### 3.1.1 Yield Parameters

**FIGURE 4:**
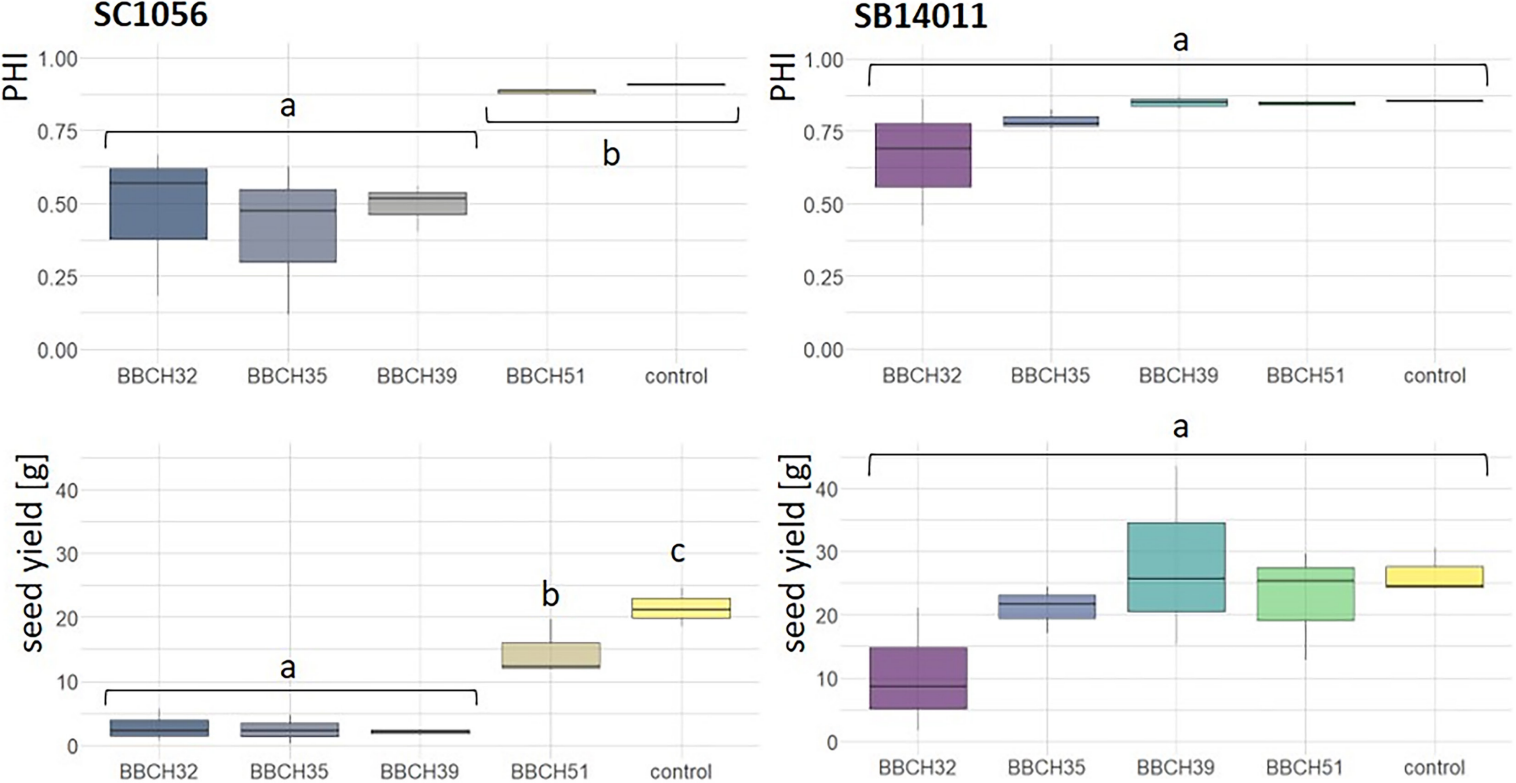
Box plots depicting the panicle harvest index (PHI) and seed yield of a cold‐tolerant (SB14011) and a cold‐sensitive (SC1056) sorghum inbred line subjected to cold stress at developmental stages BBCH32, BBCH35, BBCH39, and BBCH51 and under optimal conditions (control); *p*‐values in the Supporting Information (Table S2).

The impacts of cold stress varied significantly depending on the treatment stage and genotype (Figure 4). The cold‐sensitive inbred line SC1056 exhibited a pronounced response to cold stress at BBCH32, BBCH35, and BBCH39. Both the PHI and seed yield significantly deviated from the control in this case. Upon closer examination of seed yield, a significantly lower seed yield was also observed after cold stress at BBCH51 compared with the control. In contrast, the cold‐tolerant inbred line SB14011 showed neither significant differences in seed yield nor in PHI among the different treatments (Table 1).

**TABLE 1:** ANOVA (see M and M 2.6) analyzing possible effects of the different stress treatments and descriptive statistics for both inbred lines and their reciprocal F1 hybrids regarding the traits panicle harvest index (PHI) and seed yield.

		PHI					Seed yield (g)				
	df	MS	Error	Mean	Min	Max	MS	Error	Mean	Min	Max
SB14011	4	0.021	0.001	0.799	0.425	0.870	143.24	80.78	21.74	1.64	43.48
SB × SC	4	0.045	0.036	0.769	0.227	0.903	127.71	88.46	18.79	0.54	38.88
SC1056	4	0.176**	0.028	0.632	0.120	0.909	237.31***	7.98	8.68	0.22	24.60
SC × SB	4	0.032	0.019	0.800	0.315	0.899	291.29**	38.47	18.81	0.80	38

Significance level: ***0.001; **0.01; *0.05.

**FIGURE 5:**
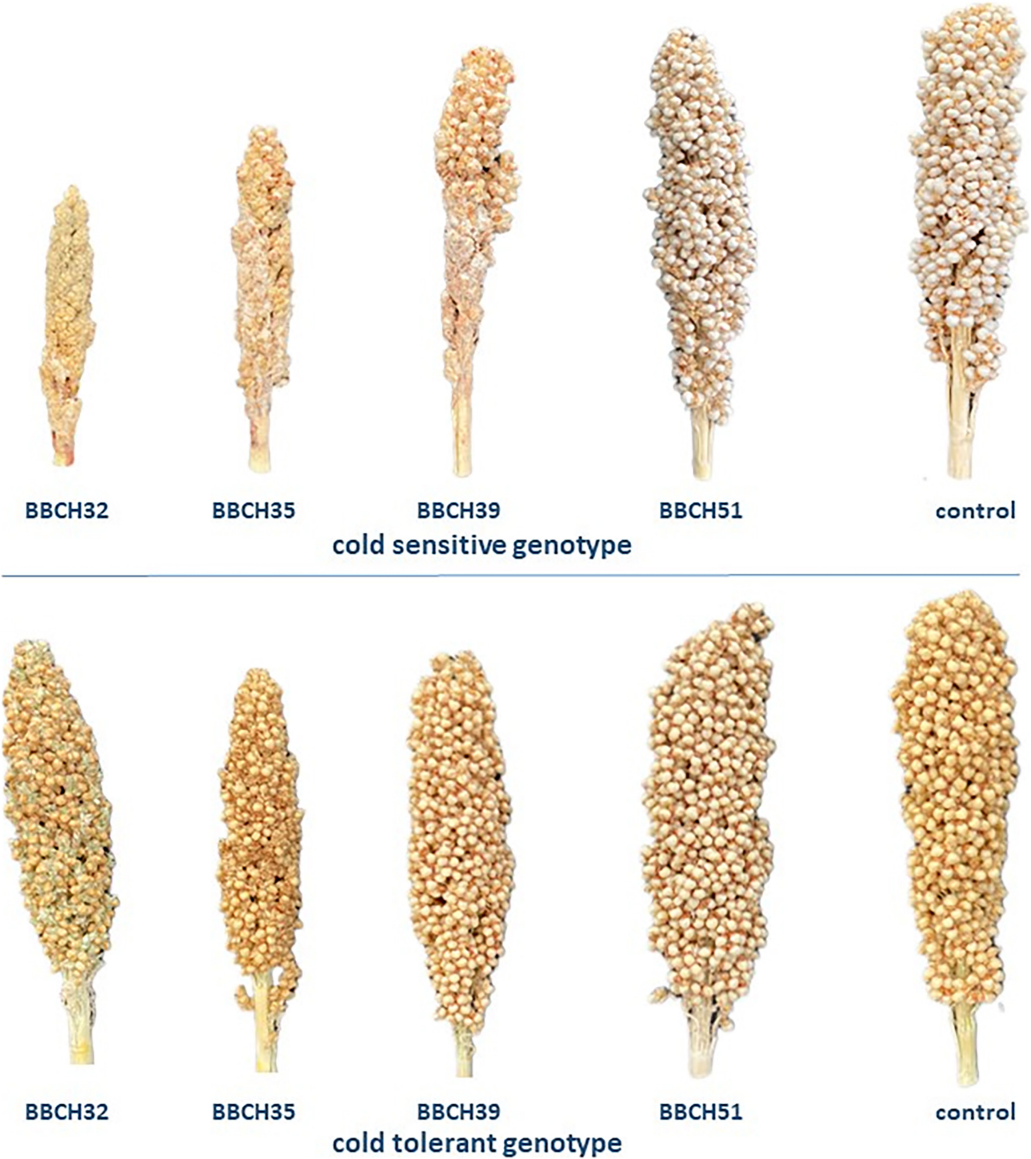
Panicles of the cold‐sensitive genotype (SC1056) and the cold tolerant genotype (SB14011) after cold stress from BBCH32, BBCH35 BBCH39, and BBCH51 and under control conditions.

The mean values of PHI and seed yield of genotype SC1056 were lowest after cold stress in the early reproductive stage (BBCH32, BBCH35, and BBCH39). For interpreting the results, it is important to highlight that the size of the panicle was also affected by cold stress (Figure 5).

**FIGURE 6:**
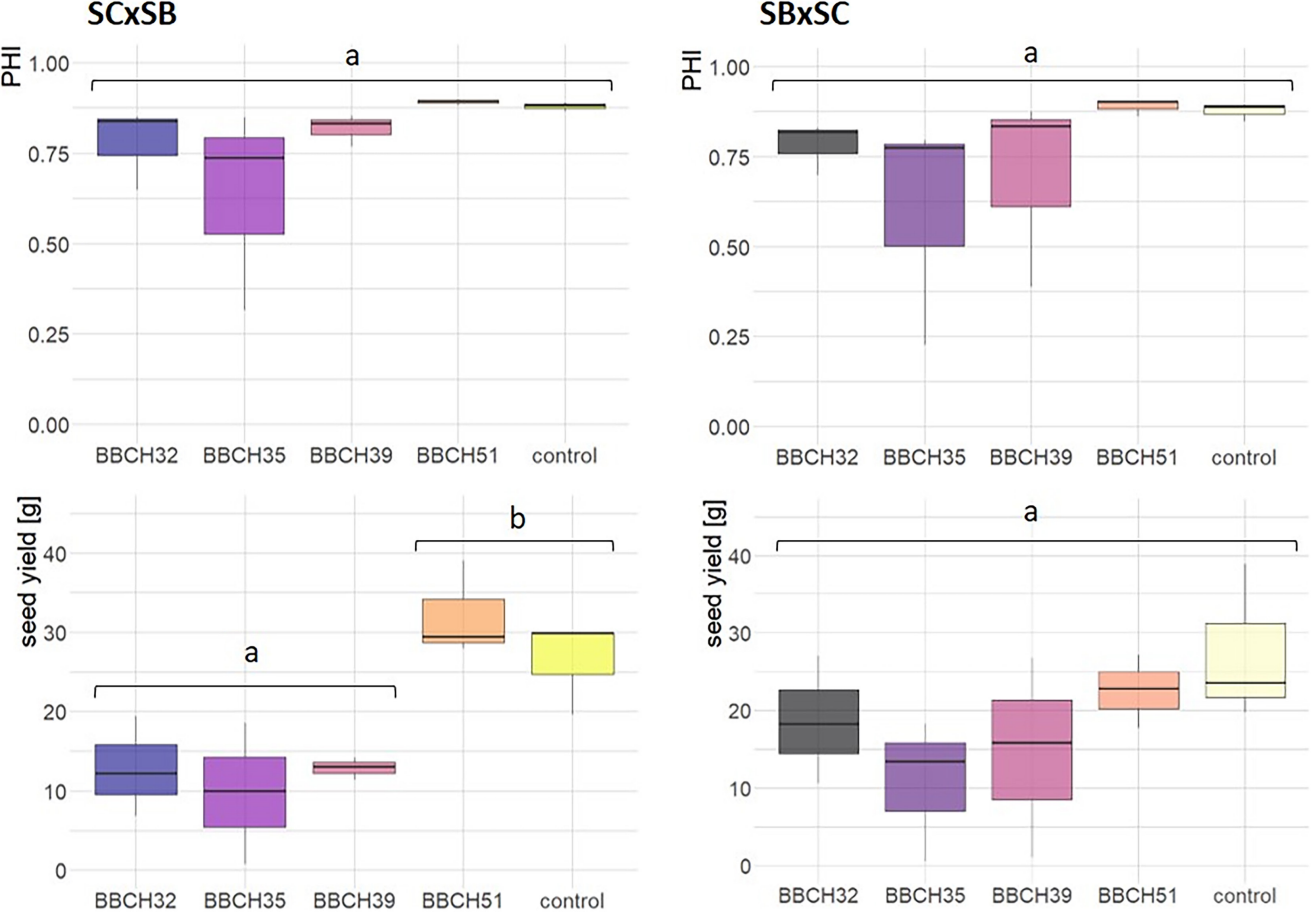
Boxplots depicting the panicle harvest index (PHI) and seed yield of the reciprocal hybrids SC1056 × SB14011 and SB14011 × SC1056 subjected to cold stress at different developmental stages (BBCH32, BBCH35, BBCH39, and BBCH51) and under optimal conditions (control); *p*‐values in the Supporting Information (Table S2).

Analyzing the impact of stress treatments on the reciprocal F1 hybrids, fewer distinctions were noted compared with the inbred lines. Only the hybrid SC × SB displayed a significantly reduced seed yield following exposure to cold stress at BBCH32, BBCH35, and BBCH39 (Figure 6), while for hybrid SB × SC, the stress treatments did not produce significant effects.

#### 3.1.2 Pollen Traits

Significant differences among various treatment levels within each genotype were found for nearly all parameters when analyzing pollen characteristics. The only exception was the proportion of fertile pollen, where no significant differences were observed between the treatments in the SC × SB hybrid. Particularly remarkable were the substantial variations in the proportion of fertile pollen between the SC1056 genotype and the F1 hybrid SC × SB, with values ranging from approximately 3% to 93% across all treatments (Table 2).

**TABLE 2:** ANOVA (see M and M 2.6) analyzing possible effects of the different stress treatments and descriptive statistics for both inbred lines and their reciprocal F1 hybrids regarding the traits proportion of fertile pollen (%), cell concentration (cells/mL), and total number of fertile pollen (cells/mL).

		Proportion of fertile pollen					Cell concentration					Total number of fertile pollen				
	df	MS	Error	Mean	Min	Max	MS	Error	Mean	Min	Max	MS	Error	Mean	Min	Max
SB14011	4	1194***	123	75.08	19.17	94.69	42 M***	2 M	3741	742	9782	29 M***	1.5 M	2885	142	8003
SB × SC	4	449.9**	115.6	77.52	41.12	94.62	23 M***	1.5 M	3432	1261	7356	14 M***	1.3 M	2707	625	5917
SC1056	4	2441.5**	255.4	70.58	3.37	92.99	12 M***	1.7 M	3031	4	6260	8.2 M***	1.4 M	2273	0.135	5480
SC × SB	4	400.4	186.2	74.64	3.55	92.68	9 M***	1.8 M	3247	20	7555	7.4 M***	1.4 M	2508	0.71	6830

Significance level: ***0.001; **0.01; *0.05.

**FIGURE 7:**
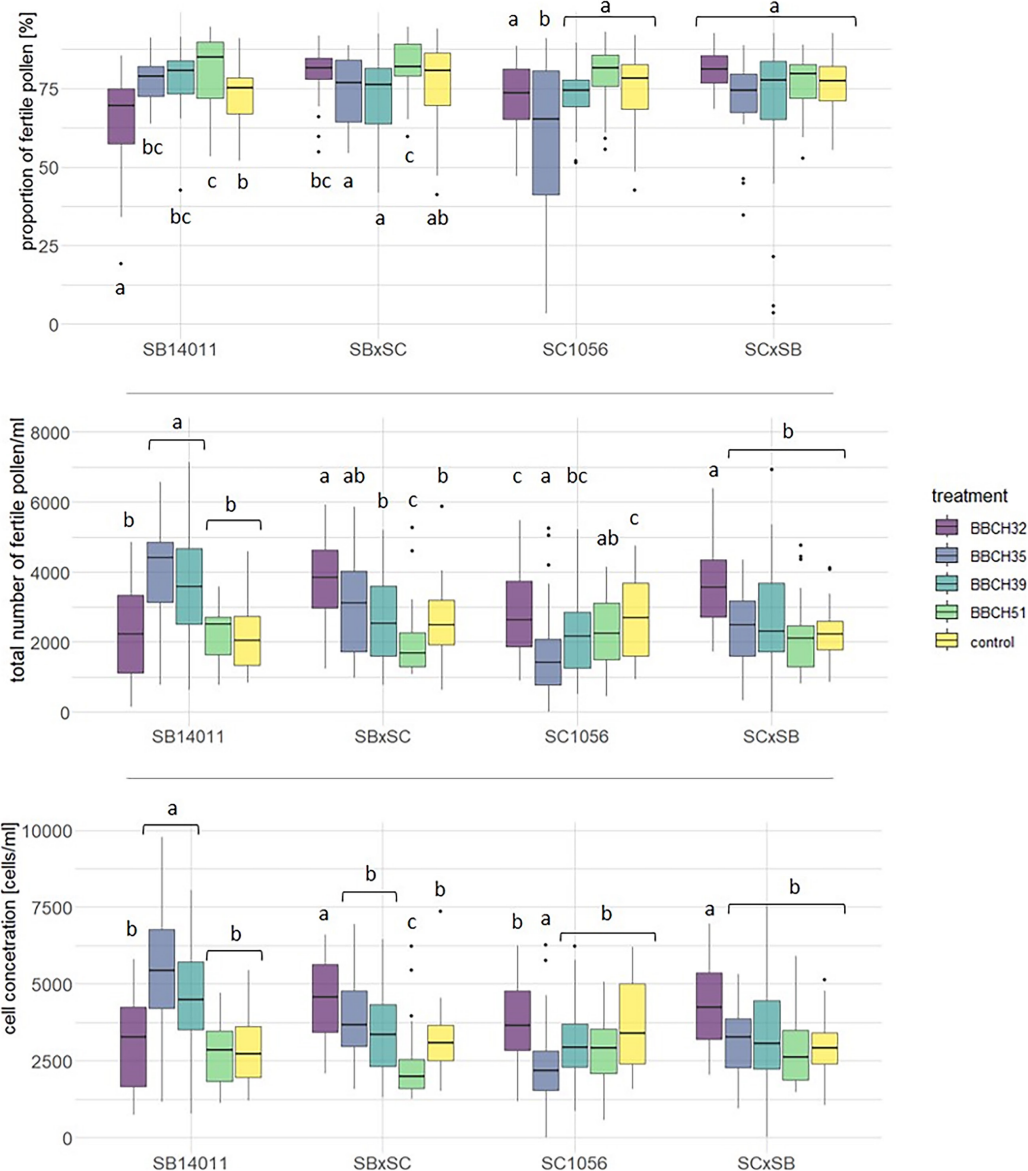
Boxplots depicting the proportion of fertile pollen, cell concentration, and total number of fertile pollen of a cold‐tolerant (SB14011), a cold‐sensitive (SC1056) sorghum inbred line and their reciprocal F1 hybrids (SB × SC and SC × SB) subjected to the different treatments; *p*‐values in the Supporting Information (Table S2).

Upon examination of the pollen characteristics in Figure 7, it became evident that the proportion of fertile pollen significantly differed from the control after cold stress at BBCH32 and BBCH51 in the cold‐tolerant genotype SB14011. In the hybrid SB × SC and the cold‐sensitive genotype SC1056, only the proportion of fertile pollen after cold stress at BBCH51 and BBCH35, respectively, differed from the control. In the SC × SB hybrid, no differences were observed between the stress treatments and the control. When cell concentration and the total number of fertile pollen were considered, significant differences were observed between various treatments and the control in all genotypes. After cold stress at BBCH32, the two reciprocal hybrids exhibited higher cell concentration and a greater total number of fertile pollen compared with the control. The same phenomenon was observed in the two parental lines after cold stress at BBCH35. SB14011 additionally showed higher cell concentration and a greater total number of fertile pollen than the control after cold stress at BBCH39. The SB × SC hybrid exhibited lower cell concentration compared with the control after cold stress at BBCH51, while the total number of fertile pollen remained at a similar level to the control.

#### 3.1.3 Genotype × Treatment Interaction

**FIGURE 8:**
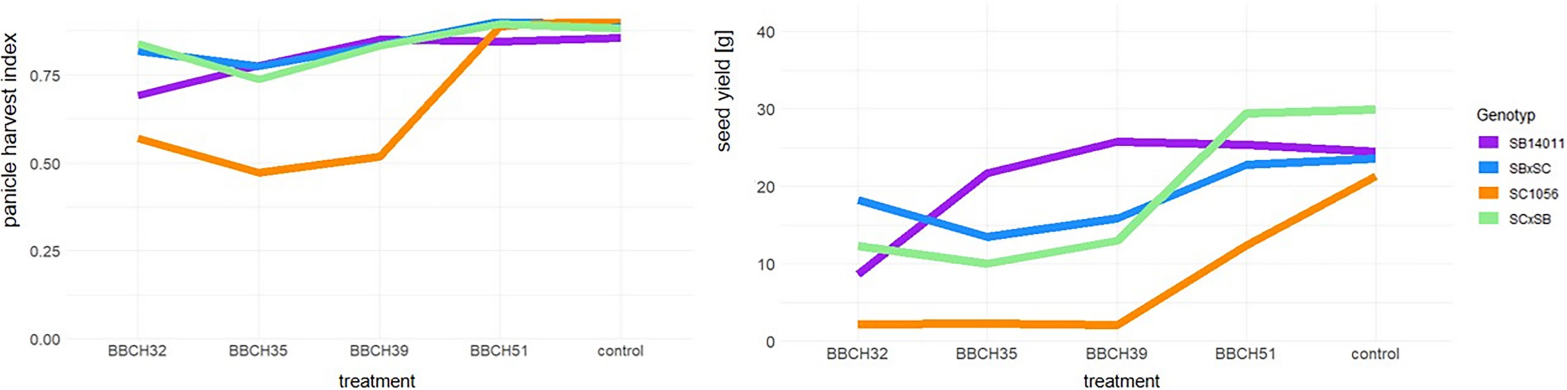
Representation of the genotype × treatment interaction for the examined features panicle harvest index (PHI) and seed yield (g); Treatment means cold stress after BBCH32, BBCH35, BBCH39, and BBCH51 and under controlled conditions. Genotypes means the cold tolerant genotype SB14011 and the cold‐sensitive genotype SC1056 as well as their reciprocal hybrids SB × SC and SC × SB.

In the examination of yield parameters, it became evident that the ranking of individual treatments in terms of PHI and seed yield was quite similar across all genotypes (Figure 8). In terms of PHI, all genotypes generally exhibited the lowest values following cold stress at BBCH32 and BBCH35, with the control achieving the highest values. A notable increase was observed from BBCH39 onwards in all genotypes. The PHI after cold stress at BBCH51 was relatively consistent across all genotypes. For seed yield, a similar pattern emerged. These yield parameters underscored the substantial difference in the early reproductive stages (BBCH32, BBCH35, and BBCH39) of the cold‐sensitive genotype SC1056 in comparison to the other genotypes. Additionally, it was noteworthy that the seed yield after cold stress at BBCH51 was significantly lower compared with the other genotypes and the control.

In summary, when assessing yield parameters, a consistent trend was observed across different genotypes with variations primarily dependent on the timing and duration of cold stress.

**FIGURE 9:**
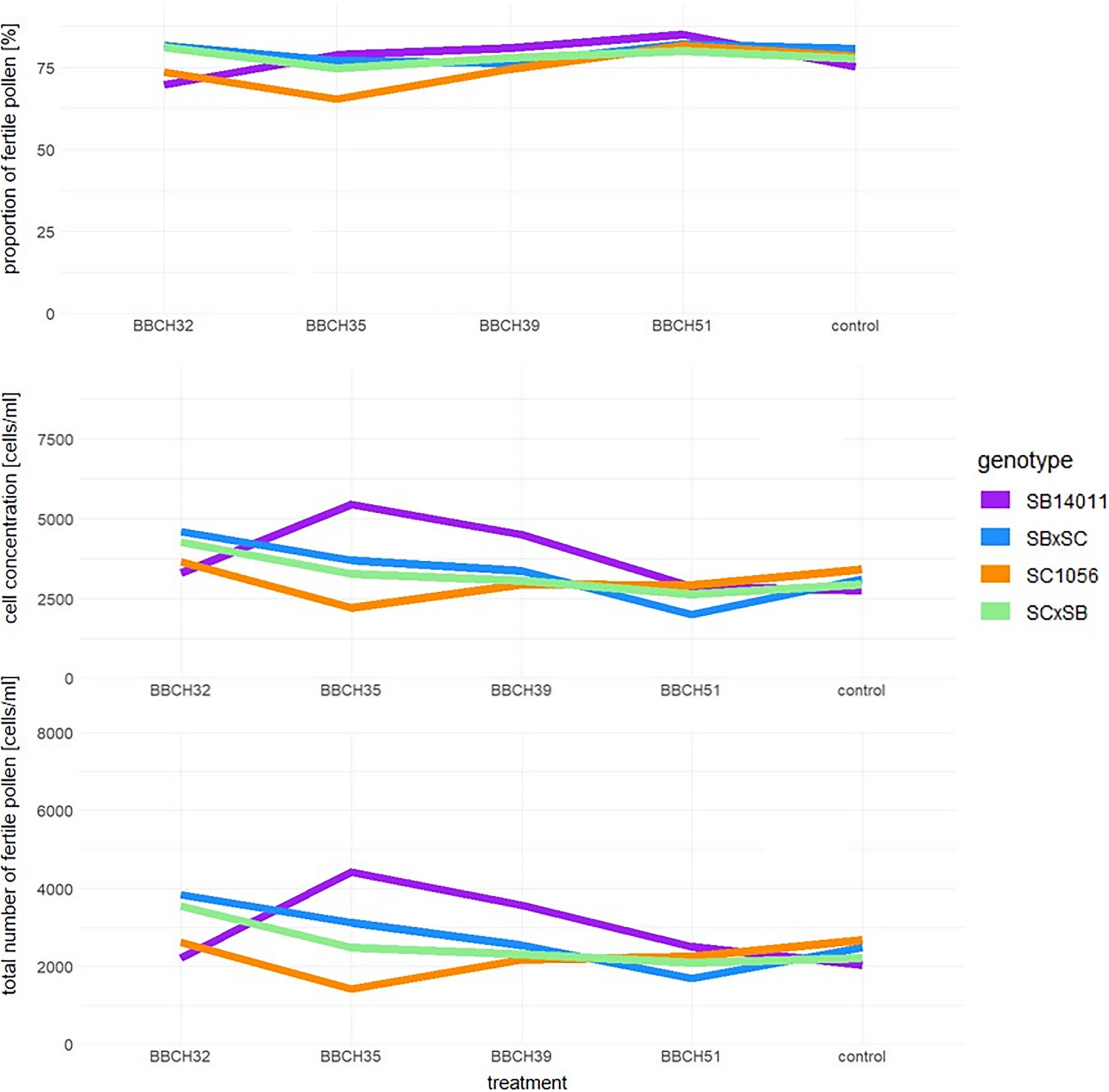
Representation of the genotype × treatment interaction for the examined features proportion of fertile pollen (%), cell concentration (cells/mL), and total number of fertile pollen (cells/mL); treatment means cold stress after BBCH32, BBCH35, BBCH39, and BBCH51 and under controlled conditions. Genotypes means the cold tolerant genotype SB14011 and the cold‐sensitive genotype SC1056 as well as their reciprocal hybrids SB × SC and SC × SB.

Figure 9 shows that the proportion of fertile pollen followed a similar ranking as observed in relation to the PHI. However, for the other two pollen traits, namely, cell concentration and the total number of fertile pollen, a somewhat different ranking became apparent, with noticeable differences between the two parental lines and their reciprocal hybrids. In the case of the cold‐tolerant genotype SB14011, there was a significant increase in both traits after cold stress at BBCH35, while the cold‐sensitive genotype SC1056 exhibited the lowest values. The two hybrids displayed a remarkably similar pattern concerning cell concentration and the total number of fertile pollen.

The cell concentration was higher the earlier the plants were exposed to cold stress. This is evidenced by the fact that the highest values were observed in plants that were subjected to cold stress from the BBCH32 stage, while the lowest values were observed in plants exposed to cold stress from the BBCH51 stage.

There was no significant genotype × treatment interaction concerning the two yield parameters, PHI and seed yield. In contrast, for all three pollen traits (proportion of fertile pollen, cell concentration, and total number of fertile pollen), a significant genotype × treatment interaction was observed (Table 3).

**TABLE 3:** Results of a multifactorial ANOVA to investigate significant differences between treatments and genotypes, as well as genotype × treatment interactions, with respect to panicle harvest index (PHI), seed yield (g), proportion of fertile pollen (%), cell concentration (cells/mL), and total number of fertile pollen (cells/mL).

	PHI			Seed yield		Proportion of fertile pollen		Cell concentration		Total number of fertile pollen	
	df	ms	Error	ms	Error	ms	Error	ms	Error	ms	Error
Genotype	3	0.096*	0.023	490.2	53.9	0.1389***	0.017	14 M***	1.7 M	11 M***	1.4 M
Treatment	4	0.182***		549.2		0.1666***		26 M***		13 M***	
G × T	6	0.031		83.5		0.0939***		19 M***		15 M***	

Significance level: ***0.001; **0.01; *0.05.

### 3.2 Correlations Between Yield Parameters and Pollen Traits

**FIGURE 10:**
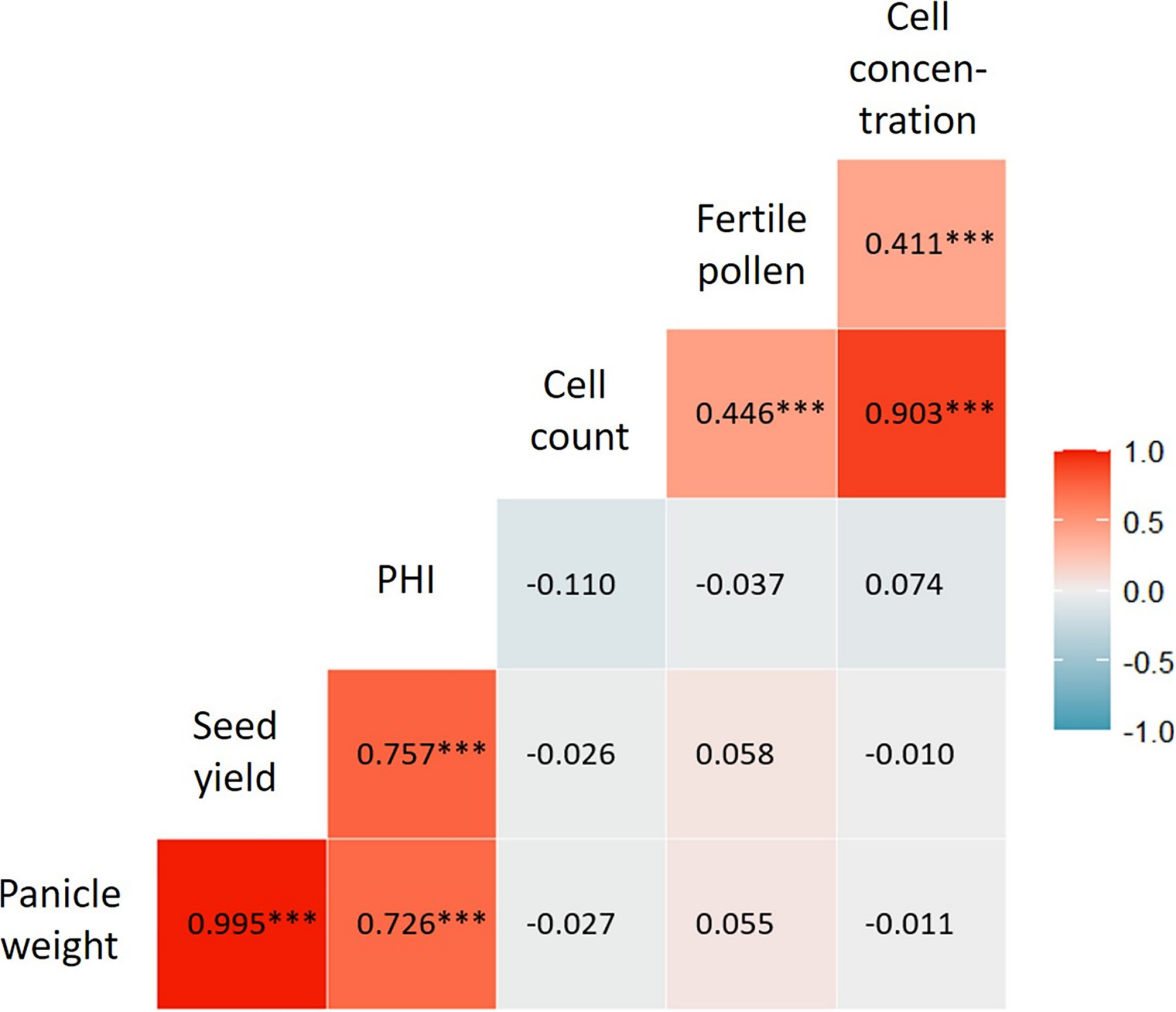
Heatmap illustrating Pearson's correlations among the considered traits: panicle weight (g), seed yield (g), panicle harvest index (PHI), cell count, fertile pollen (%), and cell concentration (cells/mL); strong positive correlations (1) are depicted in red, while strong negative correlations (−1) are represented in blue (significance level: ***0.001; **0.01; *0.05).

Pearson's correlation was calculated to investigate the presence of any correlation between yield and pollen traits. The results are presented in Figure 10.

No significant correlation was observed between any of the collected yield parameters (PHI, panicle weight, and seed yield) and the pollen traits (cell concentration, proportion of fertile pollen, and cell count). As expected, the correlation between (total) panicle weight and seed yield was very high (*r* = 0.995***), as well as between cell count and cell concentration (*r* = 0.903***). Additionally, PHI displayed strong correlations with panicle weight (0.726***), seed yield (0.757***), and the proportion of fertile pollen with both cell count (0.446***) and cell concentration (0.441***).

### 3.3 Parental Lines Versus Reciprocal F1 Hybrids

**FIGURE 11:**
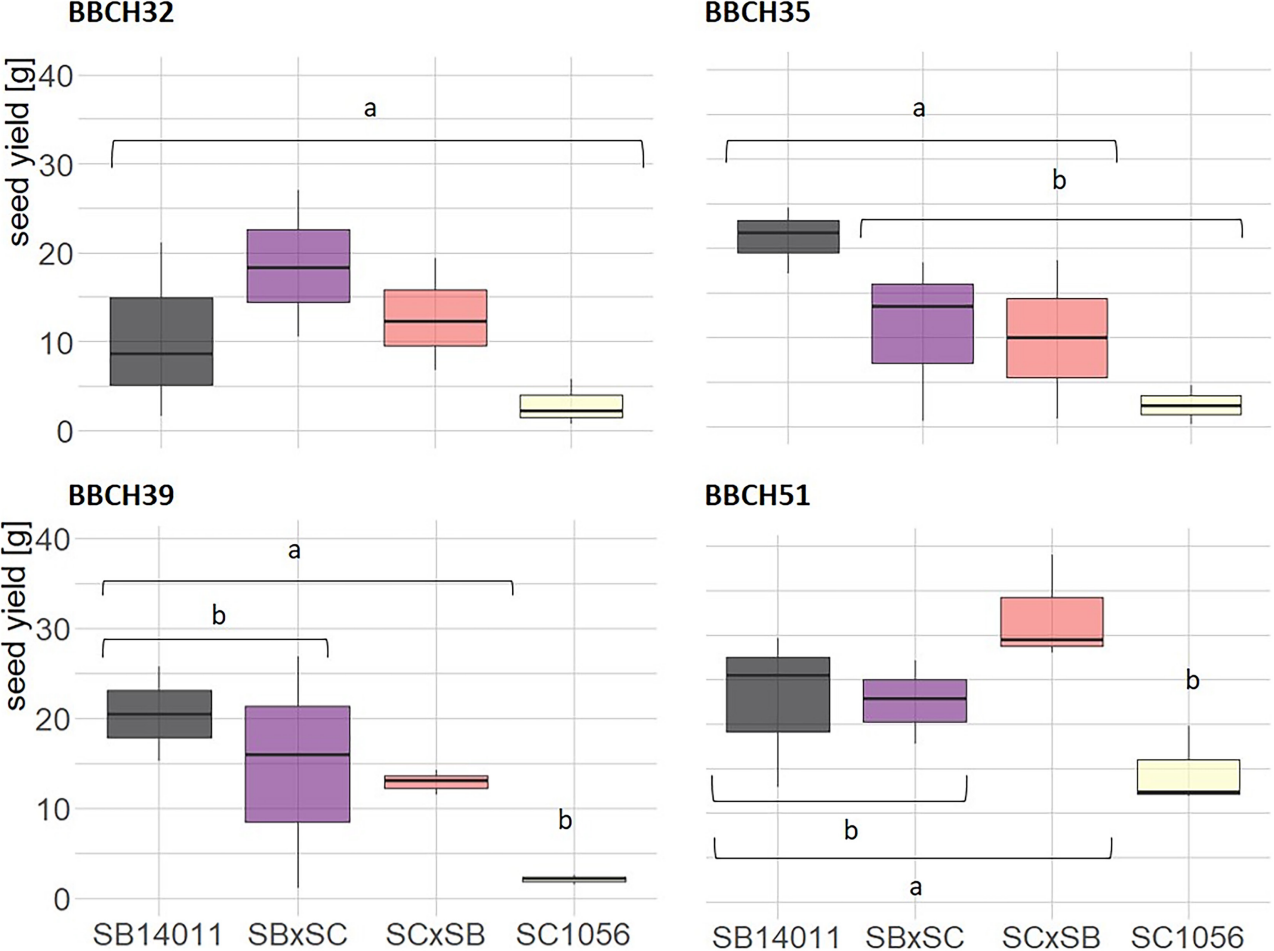
Boxplots showing the seed yield of the cold‐tolerant parental line SB14011 and the cold‐sensitive parental line SC1056 and their reciprocal F1 hybrids in the different treatment stages (cold stress after BBCH32, BBCH35, BBCH39, and BBCH51); *p*‐values in the Supporting Information (Table S1).

The *t*‐test comparing genotypes within a certain treatment revealed significant differences after cold stress at BBCH35, BBCH39, and BBCH51, and in the control in terms of at least one of the two yield parameters. At BBCH32 and BBCH35, no significant differences in PHI were observed after cold stress (Figure S1). However, when considering grain yield, significant differences were found between the hybrid SB × SC after cold stress from BBCH32 and between the two parental lines after cold stress from BBCH35. While both parental lines significantly differ from each other in terms of PHI and grain yield after cold stress from BBCH39, the hybrid SC × SB only exhibits a significantly higher PHI compared with the cold‐sensitive parental line. After cold stress from BBCH51, the cold‐tolerant parental line SB14011 significantly differs from all other genotypes in terms of PHI. When considering seed yield, only a significant difference is observed between the hybrid SC × SB and the cold‐sensitive parental line SC1056. No significant differences in seed yield were observed in plants grown under control conditions. However, significant differences were found when comparing the two parental lines and when comparing the cold‐tolerant parental line SC1056 with the hybrid SB × SC in terms of PHI. Figure 11 illustrates the differences in the yield parameter seed yield at different treatment stages. In addition to the significant differences, substantial variability within a genotype is also evident.

### 3.4 Self‐Pollination Versus Cross‐Pollination

When comparing the two pollination methods, a significant difference was observed only in the PHI of cold‐sensitive genotype SC1056 after cold stress at BBCH39 (Table 4). In terms of seed yield, only self‐pollination and cross‐pollination differed significantly after cold stress from BBCH35 of the cold‐tolerant genotype SB14011. For all other genotypes and at all treatment stages, no differences in either PHI or seed yield were found between the two pollination methods. The same result was obtained in a one‐way ANOVA pooling all genotypes and stress treatments.

**TABLE 4:** The result of the *t*‐test for the difference between the two forms of pollination (cross‐pollination vs. self‐pollination) in terms of panicle harvest index (PHI) and seed yield (g).

	BBCH	PHI	Seed yield
		*p*‐value	*p*‐value
SB14011	BBCH32	0.624	0.844
	BBCH35	0.132	0.0262*
	BBCH39	0.431	0.442
	BBCH51	0.081	0.623
SB × SC	BBCH32	0.661	0.927
	BBCH35	0.559	0.553
	BBCH39	0.518	0.733
	BBCH51	0.818	0.065
SC1056	BBCH32	0.753	0.719
	BBCH35	0.867	0.879
	BBCH39	0.015*	0.093
	BBCH51	0.534	0.912
SC × SB	BBCH32	0.993	0.321
	BBCH35	0.717	0.885
	BBCH39	0.200	0.111
	BBCH51	0.442	0.199

Significance level: ***0.001; **0.01; *0.05.

**FIGURE 12:**
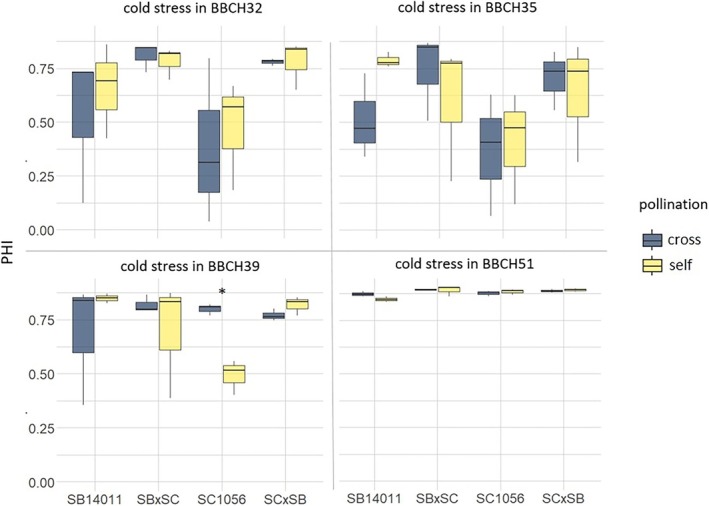
Boxplots showing the panicle harvest index (PHI) after cross‐pollination and self‐pollination of a cold‐tolerant (SB14011) and a cold‐sensitive inbred line (SC1056) and their reciprocal hybrids (SB14011 × SC1056 and SC1056 × SB14011) exposed to cold stress at different developmental stages; The significance levels refer to the differences between the pollination forms of a genotype within a treatment stage (significance level: ***0.001; **0.01; *0.05).

Figure 12 illustrates that a significant difference due to pollination methods was only found for SC1056 at BBCH39. In the cold‐sensitive inbred line SC1056, cross‐pollination at that stage led to a markedly increased PHI, whereas in the cold‐tolerant inbred line SB14011 at BBCH35, the response was the opposite, with cross‐pollination apparently reducing the PHI (not significant).

Yet, it should to be acknowledged that after cold stress in BBCH32, BBCH35, and BBCH39, the scatter of the measured values in relation to PHI is high in some cases, regardless of pollination method. After cold stress in BBCH51, the PHI is consistently high for all genotypes, with a low scatter.

## 4 Discussion

### 4.1 Cold Sensitivity in the Reproductive Development

To identify the most cold‐sensitive phases of reproductive development in sorghum, a cold‐tolerant parental line (SB14011), a cold‐sensitive parental line (SC1056), and their reciprocal F1 hybrids were exposed to cold stress from different stages of reproductive development onwards. The stages BBCH32, BBCH35, BBCH39, and BBCH51 were examined in detail. Using this experimental approach, the respective cold stress treatments differed not only in the timing of stress initiation but also in the duration of cold exposure, resulting in combined effects of these two factors.

When considering the PHI of the plants stressed at different developmental stages, the cold‐sensitive genotype SC1056 was found to be most sensitive to cold stress after exposure in the early reproductive stages (BBCH32, BBCH35, and BBCH39). This confirms the assumption made by Brooking (1976) that sorghum is most sensitive to cold before and during the leptotene stage or the interphase of microspore mother cells. After onset of panicle heading the sensitivity to cold decreases, although SC1056 still remains somewhat sensitive to cold temperatures at this stage. As shown in Figure 5, cold stress led to a reduction in panicle length, with earlier and prolonged stress exposure resulting in stronger effects. These findings highlight that not only the developmental stage of the plant but also the duration of cold stress plays a crucial role in the observed effects. While differences in panicle length are compensated for by the PHI, the reduction in grain yield is primarily attributable to smaller panicles.

In the test for existing genotype × treatment interactions, only the pollen traits showed significant results. The absence of interactions regarding the yield parameters (PHI and seed yield) can be explained by the similar response of the genotypes, underlining the strong effects of cold stress in early reproductive phase. Although not all genotypes exhibited significant yield losses after cold stress during the early reproductive phase (BBCH32, BBCH35, and BBCH39), the ranking of genotypes relative to each other remained unchanged. This suggests that both the timing of stress onset and its duration are critical factors influencing cold‐induced yield losses.

### 4.2 Importance of Pollen Traits for Reproductive Cold Tolerance

In the literature, a decrease in pollen quantity and fertility in response to low temperatures is commonly reported (Brooking 1976; Osuna‐Ortega et al. 2003). However, the present study does not confirm this assumption. Only the cold‐sensitive genotype SC1056 showed a reduced total number of fertile pollen after exposure to cold stress from BBCH35 onwards. For all other treatments and genotypes, yield reductions cannot be attributed to a decrease in fertile pollen quantity. One possible reason for this result being different in the present study may be the higher level of cold tolerance in the utilized germplasm compared with Brooking (1976) and Osuna‐Ortega et al. (2003). Surprisingly, the SC × SB hybrid exhibited a higher cell concentration and an increased number of fertile pollen even after cold stress starting at BBCH32. Elevated cell concentration and total number of fertile pollen can also be observed in the cold‐tolerant line SB14011 after cold stress from BBCH35 and BBCH39, as well as in both F1 hybrids following cold stress from BBCH32. An increased production of fertile pollen in cold‐tolerant plants, as opposed to susceptible ones, has already been observed (Sharma and Nayyar 2016). A possible explanation for the ability of sorghum to sustain the development of viable pollen under cold stress may lie in the presence of genes that accumulate a low concentration of ABA (abscisic acid), while simultaneously maintaining a sufficiently high pool of bioactive gibberellins. Additionally, reduced ABA synthesis could play a role, accompanied by a simultaneous increase in ABA catabolism due to enhanced expression of ABA hydroxylation genes. This mechanism has already been demonstrated in rice varieties (Sharma and Nayyar 2016; Parish et al. 2012). A finely tuned ABA regulation in the anthers or throughout the entire pollen sac appears to be of central importance. ABA plays a key role in plant stress responses by regulating the transcription of numerous stress‐induced genes. While many species exhibit increased ABA concentrations in floral organs under abiotic stress conditions (such as cold or drought), excessively high ABA levels can impair pollen development and fertility (Saini 1997; Parish et al. 2012). However, maintaining viable pollen alone does not necessarily ensure stable yields. This is exemplified by the SC × SB hybrid, which, despite exhibiting increased pollen production, does not always achieve a proportionally higher seed set (as measured via the PHI). This may represent a short‐term compensatory response of the plant that is insufficient to stabilize overall grain formation. The extent to which other factors, such as stigma receptivity or different pollination mechanisms, contribute to this phenomenon remains to be further investigated.

Further, no correlation between yield parameters and pollen traits was found, confirming previous results of Schaffasz, Windpassinger, Snowdon, et al. (2019). One reason might be that, due to the substantial fluctuations in the proportion of fertile pollen, cell concentration, and cell count, it is challenging to establish threshold values that might be necessary for achieving complete seed set. Nevertheless, these findings also suggest pollen traits not being the crucial factor for reduced seed set after cold stress in general, except for sensitive genotypes under certain circumstances. This assumption is reinforced by the lacking effect of cross pollination (see Section 4.3 and Figure 12).

However, it should be noted that the present analysis is based on a limited number of genotypes—two contrasting inbred lines and their reciprocal hybrids. Hence, further studies are required to validate these results on a broader genetic diversity.

### 4.3 Restriction of the Female Floral Organ

The receptivity of the pistil has repeatedly been discussed as a factor influencing cold‐sensitivity in sorghum (Osuna‐Ortega et al. 2003; Thakur et al. 2010). Even in the case of other abiotic stress factors such as drought and heat stress, pollen was initially identified as sensitive to cold, although the receptivity of the pistil later turned out to be equally responsible. Initially, the higher susceptibility of pollen to oxidative stress and the associated damage compared with pistils was discussed (Djanaguiraman et al. 2018). However, recent findings have shown that both pearl millet and sorghum stigmas respond to heat stress to a similar extent and are equally sensitive (Jagadish 2020). Even under drought stress, it could be demonstrated that the male component is not primarily responsible for low yield. Following exposure to water deficiency, although an increase in sterile pollen (up to 9%) was observed, this had no significant impact on the grain formation of the sorghum plants as well (Manjarrez‐Sandoval et al. 1989).

Nonetheless, as previously outlined, pollen fertility is still cited as the primary cause of reduced yields in sorghum after cold stress. To further investigate the impact of cold on pollen fertility and pistil receptivity, the genotypes were exposed to cold stress at different reproductive developmental stages and subjected to self‐pollination versus cross‐pollination by unstressed plants serving as pollen donors.

For the cold‐tolerant inbred line SB14011 and the reciprocal F1 hybrids, cross‐pollination had no effect on seed yield and PHI across all treatments. Cross‐pollination by an unstressed pollen donor only showed an increased PHI in the cold‐sensitive genotype SC1056 after cold stress from BBCH39, with no difference in seed yield. However, because the examination of pollen traits did not record a significantly lower proportion of fertile pollen, this effect does not seem likely to be due to the external pollen donor. Instead, partially dead parts of the panicles as a response to cold stress could explain the lower PHI of the self‐pollinated plants in this case. In all other treatment stages, i.e., cold stress from BBCH32, BBCH35, and BBCH51 onwards, cross‐pollination had no effect on the PHI and seed yield of the cold‐sensitive genotype.

Altogether, this study suggests that pollen fertility is not the limiting factor for seed set after cold stress in sorghum. Instead, it appears that the female floral organ is constrained after exposure to cold. This aligns with sorghum's reactions to other abiotic stress factors as described earlier. Furthermore, an increased sensitivity of the female gametophyte to cold stress has been observed in other species, such as *Cryptantha flava* , in the form of irreversible egg cell abortion (Casper 1990), or in chickpeas, in the form of delayed egg cell maturation and increased embryo abortion (Srinivasan et al. 1999). Because the viability of the egg cell is essential for the normal functioning of the pistil (Dumas et al. 1984), it can be assumed that cold stress also affects this aspect in the examined sorghum genotypes.

However, the results of this study are based on a limited number of genotypes—two contrasting inbred lines and their reciprocal hybrids. Generalizing these results for the highly diverse crop sorghum should be done with caution. Multiple, genotype‐specific mechanisms may contribute to reproductive cold tolerance, involving both male and female organ responses. The present findings suggest that pollen fertility is not the limiting factor; however, they do not provide conclusive evidence regarding alternative key determinants or physiological mechanisms supporting the involvement of female organs. Stigma receptivity could be a crucial factor that requires further investigation. To validate this hypothesis in future studies, histological analyses, gene expression studies, and hormone analyses could provide valuable insights.

### 4.4 Evaluation of Pollen and Yield Traits as a Parameter for Assessing Cold Tolerance

In the present study, measurements of pollen fertility, cell concentration, and cell count were conducted using the impedance flow cytometer as described by Heidmann et al. (2016). The PHI and seed yield, as proposed by Krishnamurthy et al. (2014), were used as secondary measures of reproductive cold tolerance. These traits were subsequently compared with each other. The goal of this research was to find an appropriate parameter for assessing and identifying cold‐tolerant genotypes to optimize future selection in this area. As expected, the measured yield traits showed strong correlations with each other, just like the measured pollen traits. However, none of the correlations between the yield and pollen traits were statistically significant. This could also be observed in maize plants where it was found that pollen production is important for maximum grain formation, but no direct correlation between pollen production and grain yield was found (Westgate et al. 2003). In addition, this finding is in line with Schaffasz, Windpassinger, Snowdon, et al. (2019). Due to the substantial fluctuations in the proportion of fertile pollen, cell concentration, and cell count, it is challenging to establish threshold values that might be necessary for achieving complete seed set. As more reliable indicators, the PHI or grain weight, which directly reflect seed yield, were identified. Based on the finding that pollen fertility may be of less importance for reproductive cold tolerance than previously assumed, the data collected using the IFC served as complementary parameters that could reveal associations with other potential influencing factors, such as the sensitivity of the female flower organ.

### 4.5 Inheritance of Cold Tolerance

In the literature, the complex inheritance of abiotic stress tolerance, including cold tolerance, is frequently emphasized (Thomashow 1999; Sanghera et al. 2011). This complexity underscores the importance of understanding the genetic basis of these traits to enhance the breeding process effectively. To investigate the inheritance of cold tolerance more precisely, reciprocal F1 hybrids were generated through hand emasculation between the cold‐tolerant genotype SB14011 and the cold‐sensitive genotype SC1056. By this method, the F1 hybrid has the same cytoplasm as its female parent used to analyze the per se performance of traits. This would not have been the case if we analyzed maintainer × restorer F1 hybrids, using the A1‐sterile female line to produce the maintainer × restorer cross (as done in Schaffasz, Windpassinger, Friedt, et al. 2019), since then this F1 hybrid would have another cytoplasm than the fertile maintainer line used to study per se performance for pollen and seed yield traits, implying possible distorting effects.

The analyses showed that the inheritance of cold tolerance in the examined lines depends on the specific developmental stage at which the plants were exposed to cold stress, as well as the duration of exposure. After cold stress at the developmental stages BBCH 35 and BBCH 39, no differences were observed between the parental lines and their hybrids. However, the hybrid SB × SC exhibited a significantly higher yield after cold stress from BBCH 32, and the hybrid SC × SB showed a significantly higher yield after cold stress from BBCH 51 compared with the cold‐sensitive mother SC1056. Contrary to the findings of Schaffasz, Windpassinger, Friedt, et al. (2019), instead of a pronounced heterosis, a mainly additive inheritance can be observed in this hybrid combination. The additive inheritance of QTL contributing to reproductive cold tolerance has already been described in rice (Suh et al. 2010). A statement in favor of the presumed heterotic, dominant inheritance (F1 corresponding to the better parent) can be made in this hybrid combination only for cold stress from BBCH 32 onwards. However, it has to be acknowledged that the present study has only focused on a single hybrid combination, in contrast to 49 F1 hybrid combinations evaluated by Schaffasz, Windpassinger, Friedt, et al. (2019). It is widely accepted that the expression of heterosis is combination‐specific and generally tends to increase with a higher genetic distance between the parents. Both inbred lines (SB14011 and SC1056) of the present study originate from the restorer pool of the JLU sorghum breeding program, which may have limited the magnitude of heterosis in the resulting *intra*‐pool cross compared with Schaffasz, Windpassinger, Friedt, et al. (2019), where the tested hybrids were *inter*‐pool crosses. However, the results regarding the lack of high‐parent heterosis are concordant in both studies. Additionally, the present study did not reveal differences between the two reciprocal hybrids, indicating the absence of predominating maternal or paternal inheritance. From a hybrid breeding perspective, there is thus no compelling reason to primarily select for cold tolerance in one pool.

### 4.6 Outlook

For a more precise determination of the most critical phase within reproductive development, future studies with a clear distinction between onset and duration of cold stress are necessary. Additionally, phytohormone analyses could be beneficial. They may help to identify the most sensitive stage by providing insights into the phytohormone synthesis of different genotypes following cold exposure. A more detailed understanding of phytohormone responses in cold‐sensitive genotypes would enable a better identification of adaptive mechanisms in various developmental stages of cold‐tolerant genotypes. Furthermore, the role of stigma receptivity should be further investigated, as it is discussed as a potential key factor for reproductive cold tolerance. This knowledge could facilitate the future identification of potential candidate genes for reproductive cold tolerance in *S. bicolor* .

## 5 Conclusion

A deeper understanding of reproductive cold tolerance is necessary for expanding the cultivation of sorghum into temperate regions. This study suggests that pollen fertility is not the principal factor constrained by cold stress as previously assumed, pointing to a higher importance of the female organs. Nevertheless, it should be noted that the analysis included only a limited number of genotypes, which restricts the transferability of the results. Furthermore, the early reproductive phase is confirmed as the most sensitive to cold. Acclimatization after cold stress before BBCH39 in sorghum could not be observed. Additionally, there are no predominating maternal or paternal effects observed in the inheritance of reproductive cold tolerance in F1 hybrids. Future studies should include a greater genetic diversity to further investigate the role of female organs and the genetic determinants of cold tolerance.

## Author Contributions

LN planned and supervised climate chamber experiments and data collection, performed the data analysis, interpreted the results, and wrote the manuscript. NK contributed to data analysis. RS received the funding, contributed to devise the study, and edited the manuscript. BW received the funding, contributed to devise the study, and edited the manuscript. SW devised the study, interpreted the results, and edited the manuscript. All authors contributed to the article and approved the submitted version.

## Conflicts of Interest

The authors declare no conflicts of interest.

## Supporting information


**Data S1** Supporting Information.

**Figure S1** Boxplots showing the Panicle harvest index of the cold‐tolerant parental line SB14011 and the cold‐sensitive parental line SC1056 and their reciprocal F1 hybrids in the different treatment stages (cold stress after BBCH32, BBCH39, and BBCH51); p‐values in the supplementary part (Table y).

**Data S2** Supporting Information.

## Data Availability

All phenotypic data are presented in detail in supplemental file “Data_Neitzert_2024.”
