# Supplementary figures and images for: Reproductive Cold Stress in Contrasting Sorghum Genotypes: Is Pollen Fertility Really the Crucial Trait?
Source: Plant Direct. 2025 May 4;9(5):e70065. doi: 10.1002/pld3.70065 (PMC12050216; doi:10.1002/pld3.70065)

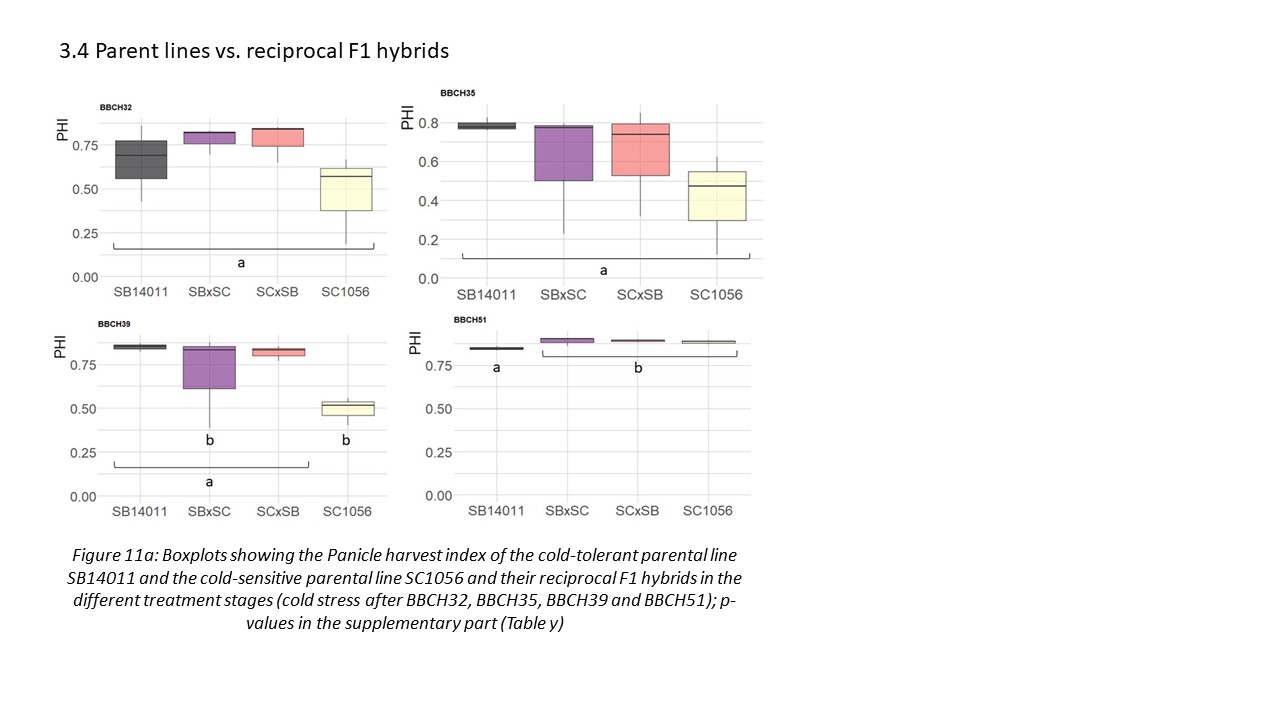

Supplement: Supplementary file 2 — Figure S1 Boxplots showing the Panicle harvest index of the cold‐tolerant parental line SB14011 and the cold‐sensitive parental line SC1056 and their reciprocal F1 hybrids in the different treatment stages (cold stress after BBCH32, BBCH39, and BBCH51); p‐values in the supplementary part (Table y). [file PLD3-9-e70065-s001.jpg]
